# Congenital Aortic Valve Stenosis

**DOI:** 10.3390/children6050069

**Published:** 2019-05-13

**Authors:** Gautam K. Singh

**Affiliations:** 1 Department of Pediatrics, Washington University School of Medicine, Campus Box 8116-NWT, 1 Children’s Place, Saint Louis, MO 63110, USA; singh_g@wustl.edu; Tel.: +1-314-454-6095; Fax: +1-314-424-2561; 2 St. Louis Children’s Hospital, 1 Children’s Place, St. Louis, MO 63110, USA

**Keywords:** congenital aortic stenosis, balloon aortic valvuloplasty, surgical aortic valvuloplasty, congenital heart disease

## Abstract

Aortic valve stenosis in children is a congenital heart defect that causes fixed form of hemodynamically significant left ventricular outflow tract obstruction with progressive course. Neonates and young infants who have aortic valve stenosis, usually develop congestive heart failure. Children and adolescents who have aortic valve stenosis, are mostly asymptomatic, although they may carry a small but significant risk of sudden death. Transcatheter or surgical intervention is indicated for symptomatic patients or those with moderate to severe left ventricular outflow tract obstruction. Many may need reintervention.

## 1. Introduction

Valvular aortic stenosis (VAS) accounts for approximately 3–6% of congenital heart defects [1]. The VAS is almost always congenital in origin in pediatric patient. It occurs more frequently in males than in females, by a ratio between 3:1 and 5:1. Associated congenital heart diseases, including patent ductus arteriosus, coarctation of aorta and ventricular septal defects, are present in 15–20% of patients who have VAS.

## 2. Anatomy and Pathology

The functional unit of a normal aortic root is the three aortic sinuses of Valsalva that are formed by an expansion of the aortic wall and the semilunar attachment of its corresponding leaflet, which create three pocket-like spaces. They are separated by commissural spaces and interleaflet triangles, the trigones [2,3]. The sum of the areas of the leaflets is greater than the cross-sectional area of the aortic root that along with tissue pliability allows for a competent closure during diastole and unobstructed valve opening during systole.

Congenital VAS displays several morphologic types of abnormal valves: unicuspid, bicuspid, tricuspid and quadricuspid [4]. The unicuspid aortic valve, most frequently seen in critical VAS, has either an eccentric orifice with one patent commissure or a central orifice with no commissure [2,4]. The single, thickened leaflet has a limited flap-valve type of opening during systole, which causes marked flow obstruction and does not lend itself well to balloon valvuloplasty [3]. The bicuspid aortic valve is the most common congenital heart disease with a prevalence of approximately 2% of the population [1]. From surgical point of view, there can be three types of morphological bicuspid aortic valve depending on number of raphe: type 0 (no raphe in the valve), type 1 (only one raphe in the valve) and type 2 (two raphes in the valve) [5]. The most common type is type 1, which accounts for about 90% of the patients with bicuspid aortic valve [6]. The two leaflets are arranged either in an anteroposterior orientation (approximately 80% of the cases) or a right–left orientation [2,7]. Valvular aortic stenosis results if the lengths of the free edges of the leaflets are less than those of the attachments to the sinuses, if the leaflets are dysplastic or if commissures are fused. The majority of bicuspid aortic valves develop degenerative changes with aging. Valvular aortic stenoses in many fetuses and infants are associated with the inadequate growth of left heart structures, left ventricular endocardial fibroelastosis and dysfunction, which constitute a distinct pathological entity. Children born with a bicuspid aortic valve have a larger ascending aorta that increases in size at a higher rate than that of matched controls with a tricuspid aortic valve [8]. There are six types of dilated aorta in children that are associated with the bicuspid aortic valve which include the normal shape (S1), the enlarged ascending aorta (S2), the effacement of the sinotubular ridge (S3), the Marfan-like (S4), the enlarged sinus of Valsalva and ascending aorta (S5), the normal annulus and proximal sinus of Valsalva, enlarged distal sinus of Valsalva, sinotubular ridge and ascending aorta (S6). S2 and S3 occur more than other types. The prevalence of dilatation at the level of the tubular ascending aorta increases with age and has been reported to be 56%, in younger than 30 years to 74%, in older than 30 years of age [9]. Both the bicuspid aortic valve and the development of bicuspid aortopathy have been attributed to genetic basis. Bicuspid aortic valve is an autosomal-dominant disease with a highly heritable trait. Autosomal dominant, X-linked and familial modes of inheritance for aortopathy have been reported. Abnormal migration of neural-crest cells probably is a common pathway that results in a bicuspid aortic valve and aortopathy [10].

Recently, the genetic basis for the pathogenesis of VAS has been identified. A study identified significantly altered CpG methylation at 59 sites in 52 genes in patients with VAS [11]. These genes are involved in positive regulation of receptor-mediated endocytosis. A significant epigenetic change in the APOA5 and PCSK9 genes known to be involved in VAS was also observed. Thus, CpG methylation can be used as molecular screening markers for the development of VAS. Some novel biomarkers that are currently being investigated such as galectin-3, growth differentiation factor-15 and micro RNAs have the potential for diagnostic and prognostic biomarkers use in VAS [12].

## 3. Pathophysiology

The pathophysiology of VAS is determined by the age of the patient at onset, its severity and any associated cardiac abnormalities.

Although the development of VAS is mostly completed by the first trimester, it may evolve throughout the gestation. Fetuses who develop severe aortic stenosis by the second trimester demonstrate failure of growth of the aortic root and left ventricle cavity [13]. Reduced growth rate of other left heart structures predict the development of hypoplastic left heart syndrome (HLHS) [13,14]. However, in isolated VAS that has associated unrestrictive foramen ovale and ductus arteriosus, the right ventricle usually compensates for the decreased left ventricular output. Valvular aortic stenosis in symptomatic neonates and young infants is usually critical in nature. The left ventricle (LV) may be limited in its ability to match cardiac output to postnatal demand because of increased afterload mismatch resulting from removal of the low-resistance placental circulation after birth. With closure of the ductus arteriosus, systemic cardiac output diminishes and congestive heart failure develops, the timing of which varies among neonates. The VAS in children and adolescents is the result of a relatively unchanged effective valve orifice with growth. The increased LV mass induced by increased wall stress initially overcompensates for pressure overload. The initial hemodynamics are characterized by normal or supranormal ejection fraction and cardiac output both at rest and during exercise [15]. Later, with progressive stenosis, the LV hypertrophy develops that causes diastolic dysfunction and increased myocardial oxygen consumption if unmatched by coronary blood flow, which may lead to myocardial ischemia and fibrosis. The diastolic dysfunction leads to increased reliance on atrial contribution to LV filling. These pathological developments may lead to stage C heart failure.

The pathogenesis of progression of stenosis at valvular level is complex. The aortic valve leaflets of VAS endure a complex mechanical environment, including leaflet stretch, fluid shear stress, bending stresses and pressure forces. Altered mechanical stimuli on the leaflets lead to valvular endothelial dysfunction, deposition of oxidized low-density lipoprotein, which may trigger infiltration of macrophages and other cytokines, resulting in inflammation. The areas of inflammation develop fibrosis, followed by leaflet thickening and finally calcification [16]. This natural progression leads to further development of stenosis and increased afterload to the LV. Although not a common feature in pediatric age, aortic aneurysm formation and aortic dissection are the two major complications of bicuspid aortopathy, mostly encountered beyond pediatric age. The aortic diameter at baseline is an important predictor of aortic expansion [17]. A recent study involving more than 400 consecutive patients with a bicuspid aortic valve showed that an aortic diameter of 40 mm or more at baseline independently predicted the subsequent development of an aneurysm, as compared with a baseline diameter of less than 40 mm in older children and adults [18]. Other predictors of progression of bicuspid aortopathy include elevated systolic blood pressure, aortic valve stenosis or regurgitation and morphologic features of the valve (R-L fusion pattern) [19]. However, the incidence of aortic dissection is low, an average of 0.1% per patient year of follow-up reported in a study of adult patients with bicuspid aortic valve [20].

## 4. Clinical Presentations

Clinical findings depend upon the age of the patient at the presentation, severity of the VAS and the presence of associated cardiac lesions.

In fetuses, VAS is diagnosed on fetal echocardiography as thicken and/or doming aortic valve with increased Doppler flow velocity >1 m/s. The VAS is a progressive lesion. Some fetal VAS may progress to HLHS manifested by the development of reversed flow in the transverse aortic arch and foramen ovale, monophasic mitral inflow and LV dysfunction in the second trimester [21]. Additionally, reduced growth rate of the left heart structures also predicts the development of HLHS [13,14].

The neonatal and well-baby check-ups perform poorly as screening tests for severe VAS. The majority of infants with severe VAS present with progressive congestive heart failure by 2 months of age. They appear pale, mottled, hypotensive and dyspneic. A normal first heart sound, an ejection click, and a gallop are present in approximately 50% of those affected. An ejection systolic murmur of variable intensity is present along the mid-left and right upper sternal borders, radiating to the carotid arteries. The presence of hypoxia (PaO2 30–40 mmHg (4–5.3 kPa)) and metabolic acidosis indicates the need for immediate medical treatment and intervention.

The older children and adolescents with VAS are usually asymptomatic. Symptoms of dyspnea, angina or syncope, particularly on exercise are usually present in 10% of older children. The development of such symptoms deserve prompt and thorough evaluation anytime because of the risk of sudden death noted in 1–10% in those between ages of 5 and 15 years of age who have moderate to severe VAS [22].

The characteristic physical findings, such as arterial pulsus parvus et tardus (small amplitude and slow-rising pulse), carotid shudder and prominent jugular venous a wave, which are described in adults with VAS, are not uniformly present in children. A precordial systolic thrill over the base of the heart is present in >65% of patients with more than mild VAS. A narrowly split, single or a paradoxically split second heart sound along with a fourth heart sound indicates severe VAS. An ejection click is present in pliable and absent in immobile aortic valve. It is unaffected by respiration and absent in severe VAS. The characteristic low-pitched crescendo–decrescendo systolic murmur of VAS, which start just after the ejection click, is best heard at the base of the heart. It radiates to the carotid arteries. The murmur of VAS increases in intensity by maneuvers that increase stroke volume, such as isotonic exercise and premature ventricular contractions.

## 5. Diagnostic Techniques

### 5.1. Lesion Morphology

**Figure 1:**
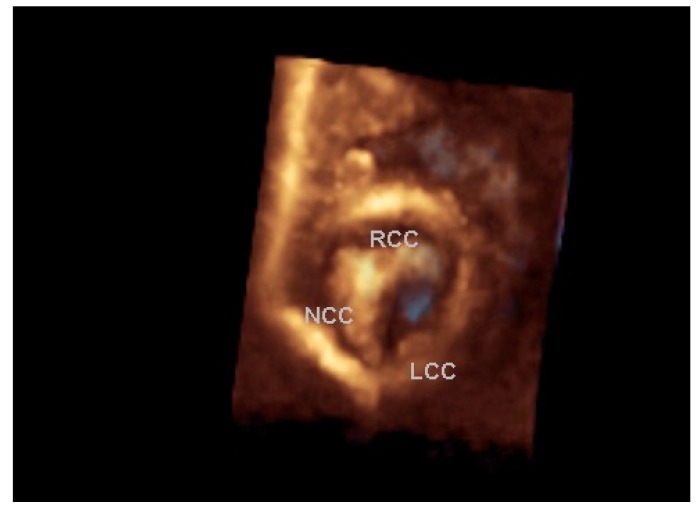
Valvular aortic stenosis with a bicuspid valve. A full volume rendered three-dimensional echocardiographic “surgeon’s view” of a bicuspid aortic valve in systole showing fused right (RCC) and noncoronary (NCC) cusps with prominent ridge, a smaller left coronary cusp (LCC) (lower to the orifice) and an eccentric elliptical restricted orifice (in light blue color).

Echocardiography is the diagnostic tool of choice in pediatric patients to evaluate valve morphology, lesion severity and LV adequacy and function. Two-dimensional and three-dimensional echocardiography demonstrate the valve morphology, aortic root dimensions, the presence of commissural fusion or raphes and the position and orientation of the valve orifice. The “surgeon’s view” (Figure 1) by three-dimensional echocardiography delineate the surgical anatomy and anatomic orifice area of the valve that can help decide the type of valve surgery or intervention. Bicuspid aortic valves due to right and left coronary leaflet cusp fusion have less risk whereas those with other types of fusion of leaflets have more risk of the occurrence of VAS and associated aortopathy. Echocardiography provides accurate morphology and aids in the classification of the types of aortic valve lesion [23]. Echocardiography also provides assessment of left ventricular hypertrophy and function and associated cardiac lesions.

### 5.2. Lesion Severity

A quantitative assessment of the severity of the valve stenosis that determines the need and timing for intervention, is estimated by aortic valve pressure gradient in pediatric patients. The transvalvular pressure gradient (P) calculated from peak instantaneous Doppler echocardiographic velocity (V) measurements obtained from multiple directions and using a simplified Bernoulli equation [24] is usually used to assess the severity of VAS in this age group:

P = 4V^2^ (1)

Based on the natural history data, in clinical pediatric practice, a catheter-measured peak-to-peak LV to aortic pressure gradient is the accepted reference for decision making to perform catheter or surgical interventions in the pediatric population [22]. However, the Doppler peak instantaneous pressure gradient overestimates the catheter-measured peak-to-peak pressure gradient. Others and we have shown that the discrepancy between the Doppler peak instantaneous pressure gradient and catheter measured peak-to-peak pressure gradient mostly occurs because Doppler gradient does not take in account the downstream pressure recovery phenomenon [25]. If the recovered pressure is deducted from the Doppler peak instantaneous pressure gradient, the net pressure reliably predicts the catheter peak-to-peak gradient [25,26]. The recovered pressure can be derived by echocardiography:

RP = 4V^2^ × 2AVA/AOA × ((1 − AVA)/AOA)) (2)

where RP is recovered pressure, AVA is aortic valve area (discussed in next section) and AOA is ascending aorta cross-sectional area [25]. The Doppler mean gradient has also been used as an estimate of the severity of VAS, because it is directly comparable to the catheter-measured mean pressure gradient. In isolated VAS, a resting peak-to-peak valve gradient (by catheter) of ≥50 mm Hg is class I indication for aortic valvuloplasty in children [27].

Because the pressure gradient, which is calculated from the measured velocity, may not correctly assess the severity of aortic stenosis in low-flow states, aortic valve area (AVA) is employed to assess accurately the severity of the lesion. It is calculated by the continuity equation. The AVA obtained by the continuity equation using echocardiography correlates closely to but often underestimates, that obtained by cardiac catheterization using the Gorlin formula [28]. In clinical pediatric practice, pitfalls in calculating left ventricular outflow tract (LVOT) area due to error in measurements of LVOT diameter make the continuity equation a less used method to evaluate the severity of VAS. Other hemodynamic measurements of the severity of VAS such as energy-loss index, AV resistance, valvulo-arterial impedance and LV stroke loss may also be calculated from the data acquired by echocardiography and catheterization [29]. However, low-flow/low-gradient and high arterial impedance are not seen in the pediatric patients and their hemodynamic measurements are not discussed here.

### 5.3. Assessment of the Adequacy of the Left Heart Structures and Function

Because adequacy of left heart structures and function influences the outcome of intervention in infants with VAS, many echocardiographic morphometric parameters and hemodynamic variables of the left heart have been used to determine whether relieving the LVOT obstruction will achieve a two-ventricle repair or whether a staged single-ventricle repair (the Norwood procedure) should be considered [30,31].

The following morphometric measurements of the left heart structures favor two-ventricle repair:

- indexed aortic annulus of ≥3.0 cm/m^2^ and indexed aortic root of ≥3.5 cm/m^2^;
- indexed mitral valve area of ≥4.75 cm^2^/m^2^;
- a ratio of the long axis of the left ventricle to that of the heart of ≥0.8; and
- left ventricular cross-sectional area of ≥2.0 cm^2^.

On the basis of some of the morphometric parameters, Rhodes et al [30]. developed a predictive equation for survival:

Discrimination Score = 14.0(body surface area) + 0.943(indexed aortic root) + 4.78(LAR) + 0.157(mitral valve area) − 12.03(3)

where LAR is the ratio of the long axis of the left ventricle to that of the heart. A discrimination score of less than −0.35 is predictive of poor outcome after two-ventricle repair.

### 5.4. Cardiac Catheterization

Cardiac catheterization for the diagnostic evaluation of VAS severity is indicated in children and adolescents with Doppler mean gradient >30 mm Hg or the peak gradient >50 mm Hg if [32]:

- they are symptomatic with angina, syncope or dyspnea on exertion (Class I);
- there is a discrepancy between clinical and noninvasive findings regarding severity of VAS (Class I);
- they are asymptomatic but have developed T-wave inversion over left precordium during exercise test (Class I);
- they are asymptomatic but are interested in athletic participation (Class I1); and
- there is several levels of LVOT obstruction in series that is likely to influence the therapeutic option or if interventional balloon aortic valvuloplasty is planned.

### 5.5. Electrocardiography

Electrocardiographic changes are neither diagnostic nor sensitive to the degree of severity of VAS in general. However, left ventricular hypertrophy with strain and ST-segment depression of 2 mm or more in the left precordial leads is a relatively sensitive indicator of severe VAS. 

There is a greater incidence of serious ventricular arrhythmias and sudden death in children and adolescents who have relatively long-standing moderate to severe VAS than in the normal population. An yearly ambulatory ECG monitoring is recommended in asymptomatic children and adolescents with Doppler mean gradient greater than 30 mm Hg or peak gradient greater than 50 mm Hg (Class I indication) [32].

### 5.6. Exercise Testing

Exercise testing is contraindicated in symptomatic patients. Graded exercise testing is a reasonable diagnostic evaluation in the children and adolescents with VAS who have a Doppler mean gradient >30 mm Hg or a peak gradient >50 mm Hg or if they are interested in athletic participation (Class II indication) [32]. Most patients who have moderate VAS but are asymptomatic have a blunted increase in systolic blood pressure (<25 mm Hg) and cardiac index during exercise [33,34]. The presence of serious arrhythmias and the extent of ST-segment depression, are indicative of myocardial ischemia and indications for therapeutic intervention [33].

## 6. Natural History

Congenital VAS is a progressive disorder. In the Second Natural History Study, the catheter measured peak-to-peak systolic gradient was found to be the most reliable indicator of the clinical course [22]. Beyond infancy, patients with a catheter-measured peak pressure gradient of less than 25mmHg have ~20% chance of requiring a valvotomy and those with a gradient of 25–49 mm Hg have ~40% chance. Patients who have a gradient of 50 mm Hg or more have a 70% chance of requiring an intervention for relief of obstruction, which, if unattended, may put them at risk of serious ventricular arrhythmia, sudden death and other morbid events at a rate of 1–1.5% per year. As with many congenital heart diseases, bacterial endocarditis is a potential complication of VAS. In the Second Natural History Study, the incidence of bacterial endocarditis was 27.1 per 10,000 person-years [35].

## 7. Management

The management of VAS is determined by the age of the patient at presentation, the severity of the obstruction and adequacy of left heart structures. The current therapeutic intervention options to relieve LVOT obstruction are percutaneous balloon aortic valvoplasty, surgical aortic valvotomy and valve replacement, all of which are intended for biventricular repair. Norwood procedure is employed for single ventricular repair.

### 7.1. Balloon Aortic Valvuloplasty

The balloon catheter is advanced retrogradely, usually via percutaneous femoral artery puncture or the umbilical artery (in the neonates), or progradely via the patent foramen ovale and is positioned across the stenotic valve over an extra-stiff exchange length guidewire. Recently, real-time 3D echocardiographic-fluoroscopic fusion imaging has allowed more efficient navigation, better visualization of the anatomic landmarks and catheter position, and more effective intervention by balloon valvuloplasty [36]. The recommended balloon:annulus ratio is 0.8–1.0. The radial dilating force exerted by the inflation of the balloon usually tears the weakest part of the valve. In bicuspid aortic valve, the balloon dilatation tears the fused commissures with adequate relief of obstruction and some valvular insufficiency. In unicuspid valve, however, balloon dilatation tends to split the leaflet opposite the patent commissure with only partial relief of obstruction and significant valvular insufficiency.

The risk of increased aortic regurgitation following balloon valvuloplasty may be significant in those who have aortic valve annulus eccentricity, which is often innate in infants and children with VAS. Measurements of different annular sizes in parasternal long axis and short axis views of 2D echo images could provide the sizes of the aortic valve annuli to help choose the appropriate size balloon catheter. It is suggested that aortic balloon valvuloplasty might help in achieving therapeutic effects when taking the different annular size into account [37]. In our experience, cardiac MRI gives an accurate way to quantify aortic regurgitation, LV volumes and function. This gives a better basis for making decision as per ACC/AHA guidelines for the need and timing of intervention in mixed lesion of VAS and aortic regurgitation. Others and we have also found circumferential as well as longitudinal strains by speckle tracking strain echocardiography as an adjunct modality for the assessment of LV function in asymptomatic patients with hemodynamically significant mixed lesions [38].

#### Immediate, Intermediate and Long-Term Results

Immediate reduction in peak pressure gradient across the aortic valve is observed in the majority of patients after balloon aortic valvuloplasty. Data from Valvuloplasty and Angioplasty of Congenital Anomalies Registry, revealed an average reduction in transvalvular gradients of 60% and procedure-related mortality of 1.9% [39]. In a single-institution study of patients in the age group 1 month to 20 years, mid-term results showed an 8-year actuarial survival of 95%, freedom from operation of 70% and freedom from intervention of 50% [40]. In an intermediate-term follow-up in a series of neonatal balloon valvuloplasties, mortality was 12% and probability of survival and freedom from reintervention at 8 years were 88% and 64%, respectively [41]. Predictors of restenosis are age 3 months or less and an immediate post-valvuloplasty peak gradient 30 mm Hg or more [39,42]. Long-term follow-up data suggest that the incidence of residual obstruction is low but nearly 25% of the patients develop restenosis, requiring reintervention [26]. Degree of immediate post-valvuloplasty aortic insufficiency is predictive of late onset of aortic insufficiency [42,43]. Thus, the immediate post-valvuloplasty incompetence grade and transvalvar gradient are the predictors of intermediate term re-intervention. 

In one of the longest (range 0.1–23.6 years) and the largest cohort (509 children with VAS) post balloon valvuloplasty follow-ups; survival free from any aortic valve reintervention was 89 ± 1% at 1 year, 72 ± 2% at 5 years, 54 ± 3% at 10 years and 27 ± 3% at 20 years. Freedom from aortic valve replacement was 90 ± 2% at 5 years, 79 ± 3% at 10 years and 53 ± 4% at 20 years. The lower post-dilation VAS gradient and lower grade of post-dilation aortic regurgitation were associated with longer freedom from aortic valve replacement but age, era and pre-dilation AS severity were not [44]. Thus, transcatheter aortic valvuloplasty is effective for relief of congenital VAS but there are steady long-term hazards for surgical aortic valve reintervention and replacement that are independent of age at initial intervention or VAS severity.

### 7.2. Surgery

Surgical valvotomy is usually performed under cardiopulmonary bypass with cardioplegic and topical hypothermic myocardial protection. Through a transverse aortotomy, a commissurotomy and the removal of fibrous excrescence and nodules from the aortic leaflets are performed. In patients who have an essentially uncorrectable valve, a small aortic annulus or recurrent stenosis with significant aortic regurgitation, an aortic valve replacement with a prosthetic valve or an aortic valve allograft may be performed in standard fashion. Because of its proven viability, the potential for growth and the lack of need for anticoagulation, pulmonary autograft (the Ross procedure) is often used for valve replacement in many centers, although it involves a previously healthy right outflow tract and predisposes it to two valve disease and further reoperations. Further controversy about the Ross operation exists regarding late dilatation of the pulmonary autograft and regurgitation of the neo-aortic valve when the procedure is performed in the full root technique. Autograft reinforcement with a prosthetic Dacron graft may be especially useful in situations known for late autograft dilatation, namely, bicuspid aortic valve, predominant aortic insufficiency and ascending aortic enlargement. The mid- to long-term results after such adjunct technique demonstrated no late aortic root enlargement and a well-functioning autograft valve without significant aortic regurgitation [45].

Patients who have been diagnosed with bicuspid aortic valve with stenosis manifest morphological changes in valve including annular eccentricity, asymmetrical calcified leaflet, presenting different sizes of leaflets. Such patients with substantial aortic valve dysfunction, aortic root dilatation and aortic dilation >4.5 cm are likely to need replacement of the aortic valve, aortic root and ascending. In patients with isolated involvement of the aortic root, surgical options may include aortic valve and aortic-root replacement with the use of a composite valve conduit (Bentall procedure) that usually have excellent outcomes in older children and adults [46].

#### Immediate and Long-Term Surgical Results

Compared to previous surgical era when surgical valvotomy in neonates with critical carried ~50% morality [47], the surgical mortality in the neonates with isolated critical VAS has reduced to less than 8% and 10-year survival thereafter has improved up to 100% with proper patient selection [48].

### 7.3. Balloon Valvuloplasty vs. Surgical Valvuloplasty for Valvular Aortic Stenosis (VAS)

Controversy exists regarding the catheter intervention versus surgical approach as the best treatment for neonates and infants with congenital VAS. In an Australian study of 123 consecutive neonates and infants who underwent relief of congenital VAS either by balloon valvuloplasty or surgical valvuloplasty, 20-year survival was 80 ± 7% and freedom from re-intervention was 40 ± 6%. Having the relief of stenosis by balloon valvuloplasty and undergoing initial treatment as a neonate were predictive of re-intervention. Freedom from re-intervention at 5 years was 27% after balloon valvuloplasty versus 65% after surgery. Thus, after surgery, a higher proportion of patients remain free of re-intervention than after interventional catheterization and the relief of their stenosis lasted longer [49]. Similar conclusion was drawn in another study of children who underwent surgical valvuloplasty (n = 89) and balloon valvuloplasty (n = 69) in the same era from an institutional experience. Balloon valvuloplasty yielded less gradient reduction, more postprocedural aortic regurgitation and a shorter interval between initial and subsequent reintervention than did the surgical valvuloplasty [50]. In a contemporary systematic review and meta-analysis to compare survival and outcomes in children with congenital VAS of 20 studies, there was no difference between surgical valvuloplasty and balloon valvuloplasty in hospital mortality (OR = 0.98, 95% CI 0.5–2.0, *P* = 0.27, I2 = 22%) or frequency of at least moderate aortic regurgitation at discharge (OR = 0.58, 95% CI 0.3–1.3, *P* = 0.09, I2 = 54%). Kaplan–Meier analysis showed no difference in long-term survival or freedom from aortic valve replacement but significantly more reintervention in the balloon valvuloplasty group (10-year freedom from reintervention of 46% (95% CI 40–52) for BAV versus 73% (95% CI 68–77) for surgical valvuloplasty, *P* < 0.001) [51]. Although higher rates of reintervention suggest improved outcomes with surgical valvuloplasty, there were more morbidity and longer hospital stay involved in surgical valvuloplasty than in balloon valvuloplasty.

### 7.4. Specific Considerations for the Management of the Infants with Critical VAS

Neonates who have critical VAS with ductus-dependent systemic circulation, present with rapidly progressive congestive heart failure as the ductus arteriosus closes. They require aggressive treatment with prostaglandin E_1_ infusion, inotropic support, diuretics, correction of metabolic acidosis and mechanical ventilation to improve the systemic perfusion. To determine the therapeutic option for single ventricle versus two-ventricle repair, the Rhode’s equation can be used. The Congenital Heart Surgeons Society, after a large multicenter prospective study, produced a multiple linear regression equation that predicts the magnitude and the direction of the optimum pathway for a 5-year survival benefit of Norwood (staged single-ventricle repair) compared with biventricular repair [52]:

Survival benefit = intercept + b_1_ (age at entry) + b_2_ (Z score of aortic valve at sinuses) + b_3_ (grade of endocardial fibroelastosis) + b_4_ (ascending aorta diameter) + b_5_ (presence of moderate or severe tricuspid regurgitation) + b_6_ (Z score of the left ventricular length). (4)

Using their appropriate transformation factors, a positive number from the equation would favor a Norwood procedure and a negative number could favor a biventricular repair. For those selected for biventricular repair, surgical aortic valvotomy and transcatheter balloon dilatation have similar outcomes but with greater likelihood of significant aortic regurgitation with the latter and of residual stenosis with the former [53]. Multiple centers as well as our preference is for balloon aortic valvuloplasty [54]. Multiple routes can be used for balloon valvuloplasty including anterograde, transumbilical venous route and transumbilical arterial route and carotid artery cut-down [54].

### 7.5. Specific Considerations for the Management of Children and Adolescents with VAS

#### 7.5.1. Indications for Intervention

Because of good results comparable to those obtained surgically as well as due to little morbidity and mortality involved, percutaneous balloon aortic valvuloplasty is recommended in children and adolescents with catheterization peak LV-to-peak aortic gradient ~50 mm Hg or if they are [32]:

- symptomatic with angina, syncope or dyspnea on exertion (Class I);
- asymptomatic but developed ST/T-wave changes on ECG at rest or with exercise (Class I);
- asymptomatic but have a catheterization peak LV-to-peak aortic gradient > 60 mm Hg (Class I);
- asymptomatic but want to play competitive sports (Class II)

#### 7.5.2. Medical Management

Prophylaxis against infective endocarditis is not recommended for VAS by the Guidelines from AHA except for prosthetic valve in aortic position [55]. The VAS is a progressive disorder and may be associated with exercise-induced sudden death in those who have moderate to severe obstruction. ACC/AHA Task Force 2 as well as Task Force 4 recommendation for participation in sports and physical exercise are the following: mild VAS with peak-to-peak catheter gradient of <30 mm Hg (Doppler mean gradient <25 mm Hg and peak gradient <40 mm Hg), moderate VAS with peak-to-peak catheter gradient from 30 to 50 mm Hg (Doppler mean gradient from 25 to 40 mm Hg and peak gradient from 40 to 70 mm Hg) and severe VAS with peak-to-peak catheter gradient of >50 mm Hg (Doppler mean gradient >40 mmHg and peak gradient >70 mm Hg) [56,57]. Those with moderate VAS should be evaluated at least yearly by two-dimensional and Doppler echocardiography, exercise testing and ambulatory ECG. They can participate in low static/low-to-moderate dynamic and moderate static/low-to-moderate dynamic competitive sports if they have mild or no LV hypertrophy by echocardiography, absence of LV strain pattern on the ECG and normal exercise test with normal blood pressure response and have no symptoms. Those with severe VAS should be followed up closely and should not participate in competitive sports [34]. Delaying intervention in these patients may not be advantageous [10]. For children and adolescents with residual moderate or severe stenosis, the recommendations for follow-up and participation in sports are same as those for untreated patients [56,57].

## 8. Prognosis

In the Second Natural History Study [22], 40% of medically managed patients subsequently required surgical intervention, whereas almost 40% of the operated patients required a second operation. Most patients after successful balloon valvuloplasty or surgery remain in New York Heart Association Class I but an incidence of sudden death has been reported, mostly in those with residual lesions or endocarditis.

The results of surgical repair of VAS in pediatric patients is generally excellent, especially if no patches are involved. The primary repair of VAS with bicuspid aortic valve has enduring results up to 10 years. Those who had balloon valvuloplasty may require repeat catheter intervention or surgical intervention in significant number of patients with VAS or mixed lesions. Intervention remains the main modality of the management. Although gene mutations polymorphism and cellular signaling pathway have been implicated in malformations of valves, none has been confirmed to play central role that restricts the clinical translational applications at the current time.

## References

[1] Hoffman J.I.E., Christianson R. (1978). Congenital heart disease in a cohort of 19,502 births with long-term follow-up. Am. J. Cardiol..

[2] Angelini A., Ho S.Y., Anderson R.H., Devine W.A., Zuberbuhler J.R., Becker A.E., Davies M.J. (1989). The morphology of the normal aortic valve as compared with the aortic valve having two leaflets. J. Thorac. Cardiovasc. Surg..

[3] McKay R., Smith A., Leung M.P., Arnold R., Anderson R.H. (1992). Morphology of the ventriculoaortic junction in critical aortic stenosis: Implications for hemodynamic function and clinical management. J. Thorac. Cardiovasc. Surg..

[4] Edwards J.E. (1965). Pathology of left ventricular outflow tract obstruction. Circulation.

[5] Sievers H.H., Schmidtke C. (2007). A classification system for the bicuspid aortic valve from 304 surgical specimens. J. Thorac. Cardiovasc. Surg..

[6] Sievers H.H., Stierle U., Mohamed S.A., Hanke T., Richardt D., Schmidtke C., Charitos E.I. (2014). Toward individualized management of the ascending aorta in bicuspid aortic valve surgery: The role of valve phenotype in 1362 patients. J. Thorac. Cardiovasc. Surg..

[7] Roberts W.C. (1970). The congenitally bicuspid aortic valve: A study of 85 autopsy cases. Am. J. Cardiol..

[8] Siu S.C., Silversides C.K. (2010). Bicuspid aortic valve disease. J. Am. Coll. Cardiol..

[9] Della Corte A., Bancone C., Quarto C., Dialetto G., Covino F.E., Scardone M., Caianiello G., Cotrufo M. (2007). Predictors of ascending aortic dilatation with bicuspid aortic valve: A wide spectrum of disease expression. Eur. J. Cardiothorac. Surg..

[10] Tadros T.M., Klein M.D., Shapira O.M. (2009). Ascending aortic dilatation associated with bicuspid aortic valve: Pathophysiology, molecular biology and clinical implications. Circulation.

[11] Radhakrishna U., Albayrak S., Alpay-Savasan Z., Zeb A., Turkoglu O., Sobolewski P., Bahado-Singh R.O. (2016). Genome-wide DNA methylation analysis and epigenetic variations associated with congenital aortic valve stenosis (AVS). PLoS ONE.

[12] Toutouzas K., Stathogiannis K., Latsios G., Synetos A., Drakopoulou M., Penesopoulou V., Michelongona A., Tsiamis E., Tousoulis D. (2019). Biomarkers in aortic valve stenosis and their clinical significance in transcatheter aortic valve implantation. Curr. Med. Chem..

[13] Simpson J.M., Sharland G.K. (1997). Natural history and outcome of aortic stenosis diagnosed prenatally. Heart.

[14] Hornberger L.K., Sanders S.P., Rein A.J., Spevak P.J., Parness I.A., Colan S.D. (1995). Left heart obstructive lesions and left ventricular growth in the midtrimester fetus. A longitudinal study. Circulation.

[15] Donner R., Carabello B.A., Black I., Spann J.E. (1983). Left ventricular wall stress in compensated aortic stenosis. Am. J. Cardiol..

[16] Rajamannan N.M., Evans F.J., Aikawa E., Grande-Allen K.J., Demer L.L., Heistad D.D., Simmons C.A., Masters K.S., Mathieu P., O’Brien K.D. (2011). Calcific aortic valve disease: Not simply a degenerative process: A review and agenda for research from the National Heart and Lung and Blood Institute Aortic Stenosis Working Group. Executive summary: Calcific aortic valve disease-2011 update. Circulation.

[17] Verma S., Siu S. (2014). Aortic dilatation in patients with bicuspid aortic valve. N. Engl. J. Med..

[18] Michelena H.I., Khanna A.D., Mahoney D., Margaryan E., Topilsky Y., Suri R.M., Eidem B., Edwards W.D., Sundt T.M., Enriquez-Sarano M. (2011). Incidence of aortic complications in patients with bicuspid aortic valves. JAMA.

[19] Khoo C., Cheung C., Jue J. (2013). Patterns of aortic dilatation in bicuspid aortic valve-associated aortopathy. J. Am. Soc. Echocardiogr..

[20] Tzemos N., Therrien J., Yip J., Thanassoulis G., Tremblay S., Jamorski M.T., Webb G.D., Siu S.C. (2008). Outcomes in adults with bicuspid aortic valves. JAMA.

[21] Mäkikallio K., McElhinney D.B., Levine J.C., Marx G.R., Colan S.D., Marshall A.C., Lock J.E., Marcus E.N., Tworetzky W. (2006). Fetal aortic valve stenosis and the evolution of hypoplastic left heart syndrome: Patient selection for fetal intervention. Circulation.

[22] Keane J.F., Driscoll D.J., Gersony W.M., Hayes C.J., Kidd L., O’Fallon W.M., Pieroni D.R., Wolfe R.R., Weidman W.H. (1993). Second natural history study of congenital heart defects: Results of treatment of patients with aortic valvar stenosis. Circulation.

[23] Sun B.J., Lee S., Jang J.Y., Kwon O., Bae J.S., Lee J.H., Kim D.H., Jung S.H., Song J.M., Kang D.H. (2017). Performance of a simplified dichotomous phenotypic classification of bicuspid aortic valve to predict type of valvulopathy and combined aortopathy. J. Am. Soc. Echocardiogr..

[24] Hatle L., Angelsen B.A., Tromsdal A. (1980). Noninvasive assessment of aortic valve stenosis by Doppler ultrasound. Br. Heart J..

[25] Baumgartner H., Stefenelli T., Niederberger J., Schima H., Maurer G. (1999). ‘Overestimation’ of catheter gradients by Doppler ultrasound in patients with aortic stenosis: A predictable manifestation of pressure recovery. J. Am. Coll. Cardiol..

[26] Barker P.C.A., Ensing G., Ludomirsky A., Bradley D.J., Lloyd T.R., Rocchini A.P. (2002). Comparison of simultaneous invasive and noninvasive measurements of pressure gradients in congenital aortic valve stenosis. J. Am. Soc. Echocardiogr..

[27] Feltes T.F., Bacha E., Beekman R.H., Cheatham J.P., Feinstein J.A., Gomes A.S., Hijazi Z.M., Ing F.F., de Moor M., Morrow W.R. (2011). Indications for cardiac catheterization and intervention in pediatric cardiac disease: A scientific statement from the American Heart Association. Circulation.

[28] Oh J.K., Taliercio C.P., Holmes D.R., Reeder G.S., Bailey K.R., Seward J.B., Tajik A.J. (1988). Prediction of the severity of aortic stenosis by Doppler aortic valve area determination: Prospective Doppler–catheterization correlation in 100 patients. J. Am. Coll. Cardiol..

[29] Donati F., Myerson S., Bissell M.M., Smith N.P., Neubauer S., Monaghan M.J., Nordsletten D.A., Lamata P. (2017). Beyond Bernoulli: Improving the accuracy and precision of noninvasive estimation of peak pressure drops. Circ. Cardiovasc. Imaging.

[30] Rhodes L.A., Colan S.D., Perry S.B., Jonas R.A., Sanders S.P. (1991). Predictors of survival in neonates with critical aortic stenosis. Circulation.

[31] Kovalchin J.P., Brook M.M., Rosenthal G.L., Suda K., Hoffman J.I.E., Silverman N.H. (1998). Echocardiographic, hemodynamic and morphometric predictors of survival after two-ventricle repair in infants with critical aortic stenosis. J. Am. Coll. Cardiol..

[32] Bonow R.O., Carabello B.A., Chatterjee K., de Leon A.C., Faxon D.P., Freed M.D., Gaasch W.H., Lytle B.W., Nishimura R.A., O’Gara P.T. (2006). ACC/AHA 2006 practice guidelines for the management of patients with valvular heart disease: Executive summary. J. Am. Coll. Cardiol..

[33] Driscoll D.J., Wolfe R.R., Gersony W.M., Hayes C.J., Keane J.F., Kidd L., O’Fallon W.M., Pieroni D.R., Weidman W.H. (1993). Cardiorespiratory response to exercise of patients with aortic stenosis, pulmonary stenosis and ventricular septal defect. Circulation.

[34] Cyran S.E., James F.W., Daniels S., Mays W., Shukla R., Kaplan S. (1988). Comparison of the cardiac output and stroke volume response to upright exercise in children with valvular and subvalvular aortic stenosis. J. Am. Coll. Cardiol..

[35] Gersony W.M., Hayes C.J., Driscoll D.J., Keane J.F., Kidd L., O’Fallon W.M., Pieroni D.R., Wolfe R.R., Weidman W.H. (1993). Bacterial endocarditis in patients with aortic stenosis or ventricular septal defect. Circulation.

[36] Jone P.N., Haak A., Petri N., Ross M., Morgan G., Wiktor D.M., Gill E., Quaife R.A., Messenger J.C., Salcedo E.E. (2019). Echocardiography-floroscopy fusion imaging for guidance of congenital and structural heart disease interventions. JACC Cardiovasc. Imaging.

[37] Chamberland C.R., Sugeng L., Abraham S., Li F., Weismann C.G. (2015). Three-dimensional evaluation of aortic valve annular shape in children with bicuspid aortic valves and/or aortic coarctation compared with controls. Am. J. Cardiol..

[38] Broch K., de Marchi S.F., Massey R., Hisdal J., Aakhus S., Gullestad L., Urheim S. (2017). Left ventricular contraction pattern in chronic aortic regurgitation and preserved ejection fraction: simultaneous stress-strain analysis by threedimensional echocardiography. J. Am. Soc. Echocardiogr..

[39] McCrindle B.W. (1996). Independent predictors of immediate results of percutaneous balloon aortic valvotomy in children: Valvuloplasty and angioplasty for congenital anomalies (VACA) registry investigators. Am. J. Cardiol..

[40] Moore P., Egito E., Mowrey H., Perry S.B., Lock J.E., Keane J.F. (1996). Midterm results of balloon dilation of congenital aortic stenosis: predictors of success. J. Am. Coll. Cardiol..

[41] Egito E.S., Moore P., O’Sullivan J., Colan S., Perry S.B., Lock J.E., Keane J.F. (1997). Transvascular balloon dilatation for neonatal critical aortic stenosis: Early and mid-term results. J. Am. Coll. Cardiol..

[42] Galal O., Rao P.S., Al-Fadley F., Wilson A.D. (1997). Follow-up results of balloon aortic valvuloplasty in children with special reference to causes of late aortic insufficiency. Am. Heart J..

[43] Rao P.S. (1999). Long-term follow-up results after balloon dilatation of pulmonic stenosis, aortic stenosis and coarctation of the aorta: A review. Progr. Cardiovasc. Dis..

[44] Brown D.W., Dipilato A.E., Chong E.C., Lock J.E., McElhinney D.B. (2010). Aortic valve reinterventions after balloon aortic valvuloplasty for congenital aortic stenosis. J. Am. Coll. Cardiol..

[45] Carrel T., Kadner A. (2016). Long-term clinical and imaging follow-up after reinforced pulmonary autograft ross procedure. Semin. Thorac. Cardiovasc. Surg. Pediatr. Card. Surg. Annu..

[46] Nazer R.I., Elhenawy A.M., Fazel S.S., Garrido-Olivares L.E., Armstrong S., David T.E. (2010). The influence of operative techniques on the outcomes of bicuspid aortic valve disease and aortic dilatation. Ann. Thorac. Surg..

[47] Kirklin J.W., Barratt-Boyes B.G. (1993). Cardiac Surgery.

[48] Alexiou C., Chen Q., Langley S.M., Salmon A.P., Keeton B.R., Haw M.P., Monro J.L. (2001). Is there still a place for open surgical valvotomy in the management of aortic stenosis in children? The view from Southampton. Eur. J. Cardiothorac. Surg..

[49] Siddiqui J., Brizard C.P., Galati J.C., Iyengar A.J., Hutchinson D., Konstantinov I.E., Wheaton G.R., Ramsay J.M., d’Udekem Y. (2013). Surgical valvotomy and repair for neonatal and infant congenital aortic stenosis achieves better results than interventional catheterization. J. Am. Coll. Cardiol..

[50] Brown J.W., Rodefeld M.D., Ruzmetov M., Eltayeb O., Yurdakok O., Turrentine M.W. (2012). Surgical valvuloplasty versus balloon aortic dilation for congenital aortic stenosis: Are evidence-based outcomes relevant?. Ann. Thorac. Surg..

[51] Hill G.D., Ginde S., Rios R., Frommelt P.C., Hill K.D. (2016). Surgical valvotomy versus balloon valvuloplasty for congenital aortic valve stenosis: A systematic review and meta-analysis. J. Am. Heart Assoc..

[52] Lofland G.K., McCrindle B.W., Williams W.G., Blackstone E.H., Tchervenkov C.I., Sittiwangkul R., Jonas R.A. (2001). Critical aortic stenosis in the neonate: a multi-institutional study of management outcomes and risk factors. Congenital Heart Surgeons Society. J. Thorac. Cardiovasc. Surg..

[53] McCrindle B.W., Blackstone E.H., Williams W.G., Sittiwangkul R., Spray T.L., Azakie A., Jonas R.A. (2001). Are outcomes of surgical versus transcatheter balloon valvotomy equivalent in neonatal critical aortic stenosis?. Circulation.

[54] Rao P.S., Singh G.K., Balfour I.C., Jureidini S.B., Fiore A.C. (1999). Balloon angioplasy of long-segment aortic coarctation in the neonate. J. Invas. Cardiol..

[55] Wilson W., Taubert K.A., Gewitz M., Lockhart P.B., Baddour L.M., Levison M., Bolger A., Cabell C.H., Takahashi M., Baltimore R.S. (2007). Prevention of Infective Endocarditis: A guideline from the American Heart Association Rheumatic Fever, Endocarditis and Kawasaki Disease Committee, Council on Cardiovascular Disease in the Young and the Council on Clinical Cardiology, Council on Cardiovascular Surgery and Anesthesia and the Quality of Care and Outcomes Research Interdisciplinary Working Group. Circulation.

[56] Graham T.P., Driscoll D.J., Gersony W.M., Newburger J.W., Rocchini A., Towbin J.A. (2005). Task Force 2: Congenital heart disease. J. Am. Coll. Cardiol..

[57] Van Hare G., Ackerman M.J., Evangelista J., Kovacs R.J., Myerburg R.J., Shafer K.M., Warnes C.A., Washington R.L., American Heart Association Electrocardiography and Arrhythmias Committee of Council on Clinical Cardiology, Council on Cardiovascular Disease in Young (2015). Eligibility and disqualification recommendations for competitive athletes with cardiovascular abnormalities: Task Force 4: Congenital heart disease. J. Am. Coll. Cardiol..

